# Surface Finish and Back-Wall Dross Behavior during the Fiber Laser Cutting of AZ31 Magnesium Alloy

**DOI:** 10.3390/mi9100485

**Published:** 2018-09-24

**Authors:** Erika García-López, Juansethi R. Ibarra-Medina, Hector R. Siller, Jan A. Lammel-Lindemann, Ciro A. Rodriguez

**Affiliations:** 1 Escuela de Ingenieria y Ciencias, Tecnologico de Monterrey, Monterrey 64849, Mexico; garcia.erika@itesm.mx (E.G.-L.); juansethi@itesm.mx (J.R.I.-M.); drlammel@itesm.mx (J.A.L.-L.); 2 Department of Engineering Technology, University of North Texas, Denton, TX 76207, USA; hector.siller@unt.edu

**Keywords:** coronary stent, magnesium alloys, AZ31, surface roughness, dross, laser cutting

## Abstract

Magnesium alloys are of increasing interest in the medical industry due to their biodegradability properties and better mechanical properties as compared to biodegradable polymers. Fiber laser cutting of AZ31 magnesium alloy tubes was carried out to study the effect of cutting conditions on wall surface roughness and back-wall dross. During the experiments, an argon gas chamber was adapted in order to avoid material reactivity with oxygen and thus better control the part quality. A surface response methodology was applied to identify the significance of pulse overlapping and pulse energy. Our results indicate minimum values of surface roughness (*R_a_* < 0.7 μm) when the spot overlapping is higher than 50%. A back-wall dross range of 0.24% to 0.94% was established. In addition, a reduction in back-wall dross accumulations was obtained after blowing away the dross particles from inside the tube using an argon gas jet, reaching values of 0.21%. Laser cutting experimental models show a quadratic model for back-wall dross related with the interaction of the pulse energy, and a linear model dependent on pulse overlapping factor for surface roughness.

## 1. Introduction

A coronary stent is a mesh-like tubular scaffold that is used to expand clogged arteries. Stenting is prescribed in about 60% of balloon angioplasty procedures, as it has been proven to reduce the rate of re-stenosis and the complication of acute vessel closure due to balloon-induced dissection [1,2,3]. Shortly after the stenting procedure, healing and reendothelialization are achieved. However, the functioning and performance of stents has not been fully validated beyond that point, and anticoagulation therapy and drug-releasing stents are as far as we have come in addressing these problems [4,5]. In an ideal scenario, the stent should be reabsorbed after a certain time in order to avoid the known complications associated with permanent stents, such as thrombosis and chronic inflammation [6]. Among the biodegradable materials, iron and magnesium alloys are preferred over polymers due to better mechanical properties and lower recoil after expansion [7]. Although the main applications for magnesium alloys have been in the orthopedic field [8,9] and as scaffolds [10] and surgical devices such as screws, plates, and fasteners [11], some efforts have been directed to the design and manufacture of magnesium-based coronary stents [12,13,14].

Several magnesium alloys have been studied for potential application in metallic biodegradable coronary stents (i.e., AZ31, AZ61, AZ80, ZM21, ZK61, and WE43). Table 1 summarizes the research conducted on fiber laser cutting of magnesium alloys for coronary stents.

For biodegradable stent design purposes, the main properties of interest are yield strength, ultimate tensile strength, elastic modulus, and corrosion rate under simulated body fluids [19,23,24]. Currently, the “Drug-Eluting Absorbable Magnesium Scaffold” (DREAMS^®^) developed by Biotronik (Berlin, Germany), is a coronary stent under clinical trials that is based on the WE43 magnesium alloy [25,26].

Laser cutting of coronary stents is a well-established process wherein cutting parameters such as laser power, frequency, pulse width, cutting speed gas type, and gas pressure are of great importance to enhance the quality features (i.e., dross, spatter, heat affected zone (HAZ), and surface roughness). For example, some experiments were carried out on the AZ31 magnesium alloy using a Q-switched fiber laser operating in the nanosecond regime comparing argon and oxygen gases to assist the process [18]. Their results revealed a loss of alloying compounds after the thermal laser process in a superficial layer, and the retention of these compounds after chemical etching, while the use of argon gas to assist the process resulted in separated struts. Also, some techniques have explored the reduction of the recast layer through the submersion on the material under a water film [27,28]. Muhammad et al. observed no presence of a heat-affected zone, debris, spatter, or recast when nitinol was submerged under a thin water film [28]. Demir et al. carried out experiments immersing the AZ31 Mg sheets in different liquids (i.e., alcohol, oil, and water) using a low ns-pulsed green fiber laser. The alcohol–water solution dissolved dross without liquid chemical instability, improving the quality of optimized conditions comparable to femtosecond laser source manufacturing [21].

The study reported here is focused on the manufacturability of AZ31 alloy during laser processing using pulses in the microsecond range. Experimental tests were implemented in an inert gas chamber with the aim of controlling the atmospheric oxygen content during laser cutting and using argon gas to blow away the melted particles inside the tube. This work addresses the simplification of a variety of laser cutting parameters (i.e., cutting speed, laser frequency, peak power, and pulse width) into two simple process parameters: pulse overlapping and pulse energy to study the impact into quality parameters such as back-wall dross and surface roughness. There is also a particular focus on exploring the reduction of back-wall dross based on gas blowing through the tube.

## 2. Methods and Materials

### 2.1. AZ31 Magnesium Alloy

Experiments were performed on two sets of AZ31 magnesium alloy tubes having different wall thicknesses. AZ31 magnesium alloy was etched using 1 mL of nitric acid, 20 mL of acetic acid, 60 mL of ethylene glycol, and 20 mL of distilled water. Table 2 illustrates and summarizes the microstructures and chemical compositions of the magnesium tubes. A cross section of the AZ31 tube extruded illustrates a large difference in grain size among individual grains with some manganese–aluminum particles (dark particles). Also, considerable twinning is visible due to the induced deformations.

Before tubes were laser cut, they were cleaned in a 70% ethanol and 30% distilled water solution for 5 min with an ultrasonic agitation bath. After ultrasonic cleaning, samples were dried by air blowing.

### 2.2. Fiber Laser and Experimental Setup

A fiber laser beam source IPG YLR-150/1500-QCW-AC was used to manufacture coronary stent struts. The fiber laser has a core diameter of 50 μm and it was focused using 120 mm collimator and a 50-mm focal lens resulting in a final theoretical spot size of 21 μm. However, according to the beam analysis provided for the machine supplier, the minimum radius is approximately ~32.1 μm [29]. Table 3 illustrates the main features of the laser source used on this work. The laser used on this work has four sub-modes (standalone, modulation, gate, and external (analog) power control) for continuous wave (CW) and pulse mode [30]. In our work, the CW laser source was modulated through a rectangular waveform. 

**Figure 1:**
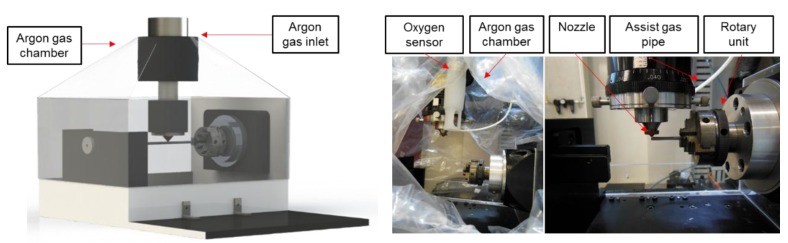
Experimental setup.

**Figure 2:**
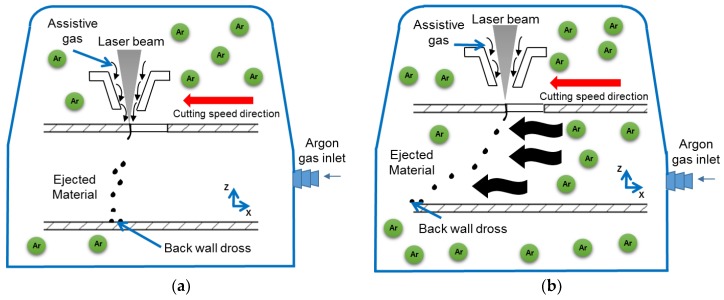
Experimental conditions under study: (**a**) Ar assistive atmosphere; (**b**) Ar assistive atmosphere and blowing through the tube.

A flexible airtight chamber made out of flexible high-density polyethylene drape was mounted on the machine (see Figure 1). It was filled with argon gas in order to create an oxygen-free atmosphere. An oxygen gas sensor (InPro 6850I from Mettler Toledo Company, Columbus, OH, USA) was used to monitor oxygen levels. All work reported in this paper was carried out at oxygen levels below 5% in the chamber. Laser cutting was performed using an assistive coaxial argon gas flow set at 4.13 bar (Figure 2a) and a standoff distance of 0.25 mm was set up between the conical nozzle and the tube surface. A surface response methodology was applied and cutting conditions were selected to evaluate the influence of blowing argon gas through the tube, when the AZ31 alloy was laser cut. This technique was implemented supplying a gas jet flowing through the inside of the tube to blow away the molten material coming down from the cutting fusion zone (Figure 2b).

### 2.3. Experimental Factors

A pulse overlapping factor *O*_f_ was used to relate periodic striations on the cut edge produced by the laser beam. This term is associated with cutting speed *v*_f_, pulse frequency *f*, and spot diameter of laser beam d by the equation [31,32]:

(1) $$\;O_{f}\;=\;100\left(1-\frac{v_{f}}{d\;\times \;f}\right)\;$$

Furthermore, pulse energy is related with peak power (*P*_peak_) and pulse width (*τ*) by the following equation:

(2) $$\;E_{p}=P_{\mathrm{peak}}\times \tau \;$$

**Figure 3:**
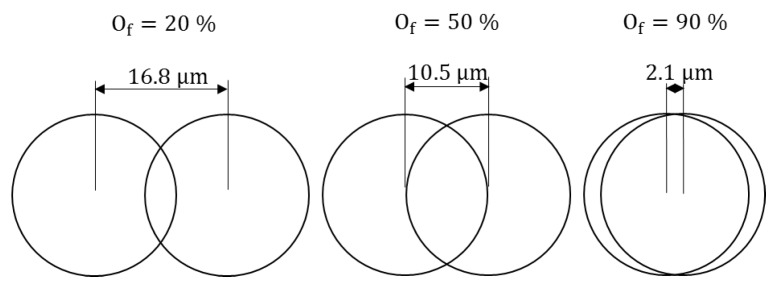
Schematic representation of pulse overlapping factor.

According to Criales et al. for the lowest pulse overlapping values (*O*_f_ < 85%), the consecutive pulses are not close enough to each other; a rough edge is obtained [32]. Therefore, an increase in the pulse overlapping causes a smoother edge. Figure 3 presents the overlap percentage between two consecutive pulses using a theoretical spot beam diameter of 21 µm.

A surface response methodology was performed to determine the influence of pulse overlapping and pulse energy on average surface roughness and back-wall dross. Separate experimental designs were applied to each tube diameter, with two replicates for pulse overlapping and pulse energy factors (see Table 4), while argon gas assisted the process (P_A_ = 4.1 bar). These process parameters were selected according to a previous experiment with laser cutting of miniature stainless steel tubes, with the same order of magnitude in wall thickness [33]. Considering that magnesium alloys have a higher thermal conductivity (~3 times) than stainless steel materials, the process parameters were selected in the same range as the cited work. From surface response methodology, two points were selected for pulse overlapping (%) and pulse energy (mJ) in which both responses were controlled. Argon gas was blown throughout the tube while a laser cut program was executed (see Table 5).

### 2.4. Response Variables

**Figure 4:**
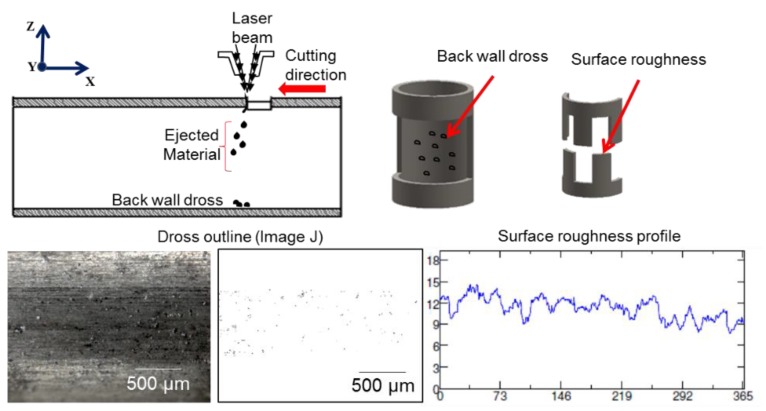
Quality-related response variables of interest.

The surface roughness was measured on the cutting edge using a confocal microscope (Zeiss Axio-CSM 700, Carl Zeiss Microscopy, LLC, Jena, Germany). Back-wall dross measurements were obtained using the following methodology. Images were acquired using a stereoscope microscope (Zeiss Discovery V8, Carl Zeiss Microscopy, LLC). The total area of the observed image was 2190 µm × 1630 µm for the tube with an outer diameter of 3 mm and thickness of 220 µm, while it was 1080 µm × 780 µm for tubes with an outer diameter of 1.8 mm and thickness of 160 µm. All images were captured in the middle of the tube because this area was the most influenced by laser trajectory. Then, images were analyzed using Image J [34] software (version 1.48, National Institutes of Health, Bethesda, MD, USA) in order to quantify the area covered by dross from the fusion zone. Dross particles images were converted to binary using a threshold function and each image was measured with the particle analysis module. In this module, a maximum particle diameter of 50 µm was selected according to the dross particles measured on the bottom area. The back-wall dross percentage was based on area covered with dross and the total measured area. Figure 4 illustrates the variables measured and the geometry that was cut.

## 3. Results

### 3.1. Cutting with No Gas Blowing through the Tube

Surface response design trials were programed following the experimental levels presented in Table 6. These levels were chosen according to the laser cutter restrictions. Experimental results are shown in Table 7 (two replications averaged for each response).

**Figure 5:**
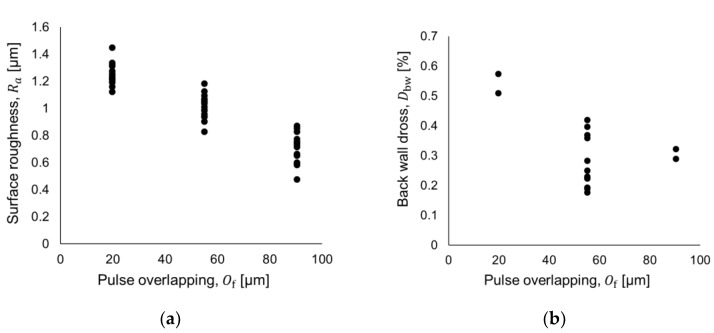
Illustration of variability in the process: (**a**) surface roughness and (**b**) back-wall dross (Tube A *OD* = 3.0 mm & *t* = 0.22 mm).

Figure 5 illustrates the process variations in surface roughness (Figure 5a) and back-wall dross responses for Tube A (Figure 5b), at different levels of pulse overlap and under pulse energy of 30.5 mJ. These plots exemplify the low dispersion of the data in spite of the surface design modification, which would have an effect on the design orthogonality. In fact, the conclusion of an orthogonal design is the basis of a study motivated by the minimization of variance [35], while our study is focused on the minimization of practical responses (i.e., back-wall dross and surface roughness).

Equations (3) and (5) present the models for surface roughness response and Equations (4) and (6) show the back-wall dross models for Tubes A and B, respectively. Even though Equations (3) and (5) are based on pulse energy, the peak power and pulse width are inherent in the process (i.e., these parameters are programmed on machine with a pulse energy range between 26.97 mJ to 34.04 mJ). Therefore, the equations are conclusive for the range of pulse energy established in Table 4.

**Figure 6:**
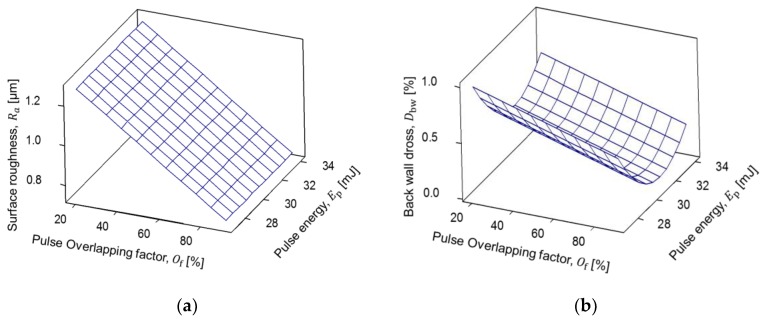
Results for Tube A (3 mm outside diameter and 0.22 mm wall thickness): (**a**) average surface roughness response; (**b**) back-wall dross response.

**Figure 7:**
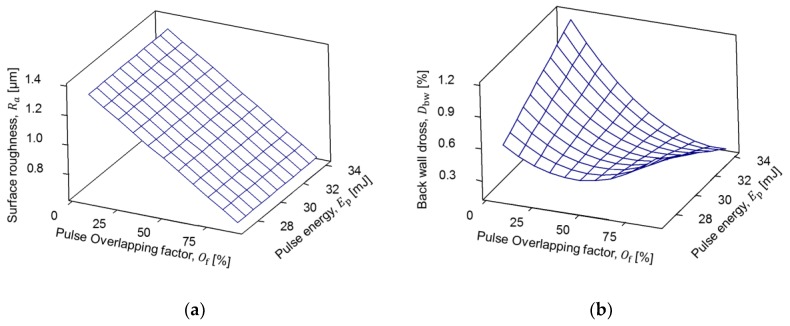
Results for Tube B (1.8 mm outside diameter and 0.16 mm thickness): (**a**) average surface roughness response; (**b**) back-wall dross response.

From the regression models, surface roughness has a linear model and is strongly dependent on the pulse overlapping parameter. Furthermore, back-wall dross has a quadratic model and is related to the quantity of energy applied to melt the surface and separate the coronary strut geometry. The results for the models are plotted in Figure 6 and Figure 7. For fiber laser cutting of coronary struts with outer diameter of 3 mm and 0.22 mm wall thickness (Figure 6a), a minimum average surface roughness of 0.7 µm was found using a pulse overlapping of 90.36% and pulse energy of 30.5 mJ.

Moreover, an average back-wall dross percentage (Figure 6b) of 0.08% was identified using the same overlap percentage but increasing the pulse energy to 31.2 mJ. Also, for fiber laser cutting of coronary struts with an outer diameter of 1.8 mm and 0.16 mm wall thickness, the minimum average surface roughness (Figure 7a) was found with a high overlap value (90.01%) in the whole range of pulse energy presented in Table 4, while a minimum back-wall dross (Figure 7b) of 0.17% was established with a pulse energy of 34.035 mJ at the same pulse overlap.

(3) $$\;R_{a}=1.01-0.187\times O_{f}\;$$

(4) $$\;D_{\mathrm{bw}}=0.277-0.139\;\times O_{f}-0.105\times \;E_{P}+0.182\times E_{P}{}^{2}\;$$

(5) $$\;R_{a}=1.0-0.24\times O_{f}\;$$

(6) $$\;D_{\mathrm{bw}}=0.408-0.159\times O_{f}+0.133\times O_{f}{}^{2}-0.135\times O_{f}\times E_{P}\;$$

**Figure 8:**
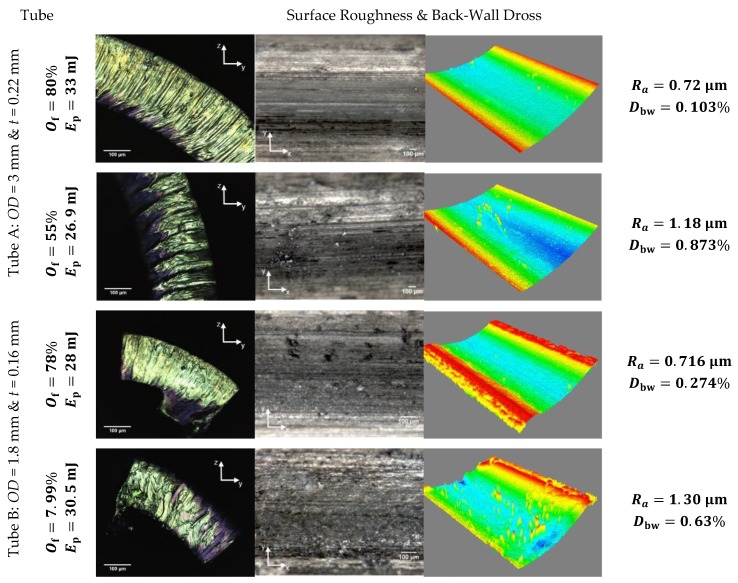
Selected process conditions and corresponding surface roughness and back-wall dross—experimentation without gas blowing through the tube.

Analysis of variance (ANOVA) test of each experimental design and determination coefficients are presented in Table 8 and significant parameters are highlighted. Figure 8 illustrates a comparison of the selected process parameters and the corresponding surface roughness and back-wall dross responses. The lowest values of pulse overlaps (less than 50%) promote a rougher and more irregular cut edge, which represents a challenge for the post-processing cleaning. In particular, the surface topography shows dross particles adhered to the surface in piles below the laser trajectory. These particles pile up, causing a different chemical etching rate when the stents are post-processed and thus a non-uniform surface.

### 3.2. Cutting Using Gas Blowing through the Tube

A preliminary study that consisted of blowing a jet of argon gas through the tube in addition to the cutting carried out in the chamber was explored as a method of blowing away molten particles being ejected from the fusion zone, and thus reducing the dross on the tube back wall.

**Figure 9:**
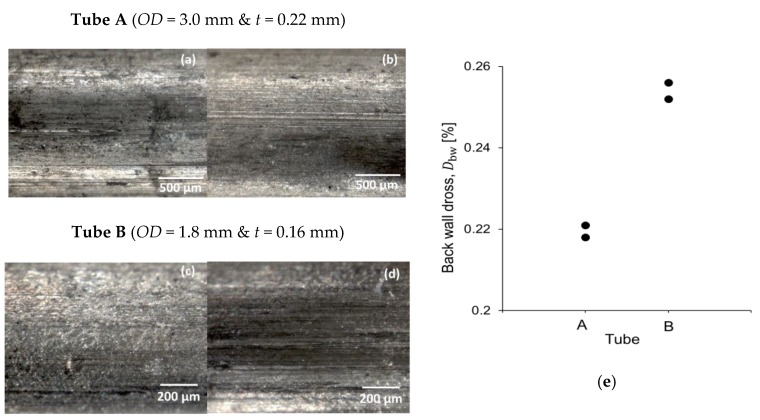
Comparison of treatment: without (**a**,**c**) and with (**b**,**d**) gas blowing through the tube. (**e**) Back-wall dross response under the condition of blowing gas inside the tube.

From the results in Table 7, one condition for each tube was selected (based on the process parameters indicated in Table 5). Our results indicate a reduction of the back-wall dross through the use of gas blowing through the tube (see Figure 9e). For example, back-wall dross values were reduced from 0.31% (without blowing gas through the tube) to 0.21% (with blowing of gas through the tube) using a pulse overlapping at 90.36% and a pulse energy of 30.5 mJ in tube A.

Figure 9a–d presents a qualitative study of the surface obtained with a stereomicroscope comparing both treatments. These results are promising and could be used for further improving stent quality in a cost-effective manner. Hence, future work will focus on studying the influence of gas flow rate and pressure inside tubes for the blowing of dross particles.

## 4. Discussion

Surface response methodology was applied to minimize surface roughness and back-wall dross response. Investigations regarding the laser cutting mechanism of magnesium and its alloys are of particular interest because of the high thermal reactivity of these materials. Several methods had been studied to minimize back-wall dross on magnesium alloys laser cutting (i.e., water, alcohol–water solution, and paraffin based oil) [21]. These methods have proven to be viable; however, the control of post-processing and the effects of these treatments on the quality assurance of coronary stents have not been studied.

Our results demonstrate the control of surface roughness and back-wall dross by carefully tuning cutting conditions. In particular, a surface roughness below 0.7 μm can be achieved increasing the pulse-overlapping factor over 50%. For back-wall dross response, considerably good results were found using the inert gas chamber. This chamber provided stability on laser cutting, by avoiding reactions between magnesium and oxygen and reducing the thermal effects. Whereas some researchers have used ultrashort-pulsed laser sources (i.e., nanoseconds, and femtoseconds) for reducing thermal effects and achieving average surface roughness below 1.4 μm [20]; our approach has achieved even lower roughness values while at the same time avoiding the high costs associated with using expensive ultra-short pulsed laser sources. For back-wall dross response, minimum values below 1.0% were observed on the central area of the cutting trajectory.

Table 2 shows a different microstructure of Tube A vs. B. This different in grain size could influence the process responses of interest in the present study. On the other hand, the difference in wall thickness for Tube A vs. B is significant. In this study, no attempt was made to draw conclusions based solely on the different material microstructures because the wall thickness plays a more significant role in terms of heat transfer conditions. All conclusions in this study were treated separately when discussing Tube A vs. B.

The effect of pulse overlapping and pulse energy on surface roughness is well documented in previous studies [36]. There is an underling geometrical connection between the pulse overlapping and the resulting surface roughness, as shown schematically in Figure 3. In this study, we provide a detailed quantification of this phenomenon for the AZ31 alloy at small wall thickness (see Figure 6a and Figure 7a).

On the other hand, the effect of pulse overlapping and pulse energy on back-wall dross is intriguing. Figure 6b and Figure 7b show a highly non-linear response. The overall tendency of reduction in back-wall dross as pulse overlapping increases is because there is simply less material to expel towards the back wall. However, the non-monotonic influence of pulse energy is puzzling. The expelled material from the cutting side of the tube will form particles in midair that adhere to the back wall. It is clear that, given the same amount of material available per unit time to expel (established by the pulse overlapping), different levels of pulse energy produce certain amount particles that do not adhere to the back wall. In the case of Tube A, there is a middle level of pulse energy that minimizes the amount of material sticking to the wall. In the case of Tube B, there is a clear interaction between pulse overlap and pulse energy. This is most likely a phenomenon governed by the thermal and physical properties of solid and molten magnesium alloys, together with the fluid mechanics at this microscopic scale [37]. Given that the literature regarding this phenomenon is scarce, more experimental and modeling research is required.

Current literature on back-wall dross effects is limited. One of the few studies discusses the removal of dross using a water stream through a stainless-steel tube [27]. Whereas this has been reported to result in an absence of back-wall damage and dross percentage reduction from 0.7% to 0.2%, water immersion for magnesium is not practical due to material reactions. In our study, blowing argon gas through the tube showed dragging effects on dross particles reducing the values obtained for back-wall dross using the same cutting conditions. For Tubes A and B, a reduction of ~29% and ~10%, respectively, was found after blowing out the melted particles. Further studies must analyze the effect of the flow rate and gas pressure inside tubing using inert gases to achieve even better quality in magnesium applications.

## 5. Conclusions

This work demonstrates the use of a microsecond quasi continuous wave (QCW) fiber laser source as a competitive option for the manufacture coronary stents in AZ31 magnesium alloy material. The conclusions can be summarized as following:

A gas chamber was implemented to provide an inert gas atmosphere during laser cutting in order to avoid the effects of material reactivity and the process variability on experiments.

Laser cuts were performed in AZ31 magnesium alloy miniature tubes with outer diameter of 3 mm and 1.8 mm and wall thicknesses of 0.22 mm and 0.16 mm, respectively. Low values of surface roughness (*R_a_* < 0.7 μm) were obtained by increasing the pulse overlapping over 50%. An increase of this overlapping is related to a reduction of the cutting speed.

Back-wall dross measurements were evaluated in the central part of the trajectory. Under normal processing conditions (no blowing of argon through the tube), a range from 0.24% to 0.94% was quantified. However, the technique of blowing argon through the rube proved to significantly improve back-wall dross, reducing it to about 0.22%.

In terms of future work, additional studies must be performed to identify the effect of gas flow rate and pressure of gas inside tubes in order to reduce back-wall dross and the thermal effects caused by the laser.

## Figures and Tables

**Table 1:** Review for fiber laser cutting of AZ31 magnesium alloy.

Ref.	Test Alloy, *OD* (*t*) *	Process Parameters		Response
[15]	AZ31 Mg, Tube *OD* = 2.5 mm (200 μm)	Laser mode	Pulsed-QCW	Surface quality
		Laser power (W)	50	
		Pulse frequency, *f* (kHz)	20–80	
		Pulse width, *τ* (ms)	0.0001	
		Gas type & pressure (bar)	O_2_, Ar (6.9)	
		Cut speed, *v*_f_ (mm/min)	120	
[16]	AZ31 Mg, Sheet (1000 μm)	Laser mode	CW	Dross height *R_a_*
		Laser power (W)	2000	
		Gas type & pressure (bar)	Ar (3–6)	
		Cut speed, *v*_f_ (mm/min)	10,000–30,000	
[17]	AZ31 Mg, Sheet (1000–3000 μm)	Laser mode	CW	Striation inclination Dross height *R_a_*
		Laser power (W)	2000	
		Gas type & pressure (bar)	Ar (3–6)	
		Cut speed, *v*_f_ (mm/min)	2000–30,000	
[18]	AZ31 Mg, Tube *OD* = 2.5 mm (200 μm)	Laser mode	Pulsed-QCW	*R_a_*
		Laser power (W)	4.5–7.5	
		Pulse frequency, *f* (KHz)	25	
		Pulse width, *τ* (ms)	0.0001	
		Gas type & pressure (bar)	O_2_, Ar (6.9)	
		Cut speed, *v*_f_ (mm/min)	120	
[19]	AZ31 Mg, Sheet (400 μm)	Laser mode	CW	*R_a_* Dross Kerf width
		Laser power (W)	150	
		Gas type & pressure (bar)	N_2_ (6)	
		Cut speed, *v*_f_ (mm/min)	1200	
[20]	AZ31 Mg, Tube *OD* = 2.5 mm (200 µm)	Laser mode	Pulsed	n/a
		Laser power (W)	7.5	
		Pulse frequency, *f* (KHz)	25	
		Pulse width, *τ* (ms)	0.0001	
		Gas type & pressure (bar)	Ar (6.9)	
		Cut speed, *v*_f_ (mm/min)	120	
[20]	AZ31 Mg, Tube *OD* = 2.5 mm (200 µm)	Laser mode	Pulsed	*R_a_* Dross Kerf width
		Laser power (W)	5	
		Pulse frequency, *f* (KHz)	200	
		Pulse width, *τ* (fs)	800	
		Gas type & pressure (bar)	Ar (6)	
		Cut speed, *v*_f_ (mm/min)	300	
[21]	AZ31 Mg, Sheet (250 µm)	Laser mode	Pulsed	Dross Kerf width Taper angle
		Laser power (W)	6	
		Pulse frequency, *f* (KHz)	300	
		Pulse width, *τ* (ns)	1	
		Cut speed, *v*_f_ (mm/min)	15–315	
[22]	MgCa Sheet (700 µm)	Laser mode	Pulsed	Kerf width Taper angle *R_a_*
		Laser power (W)	300–1500	
		Pulse frequency, *f* (KHz)	1000	
		Pulse width, *τ* (ns)	0.1–0.5	
		Gas type & pressure (bar)	Ar (8.2)	
		Cut speed, *v*_f_ (mm/min)	100–1000	

* Dimensions are given in outside diameter (*OD*) and thickness (*t*) for tube wall or for sheet.

**Table 2:** Microstructure and chemical composition of magnesium miniature tubes.

Tube A (*OD* = 3 mm & *t* = 0.22 mm)		Tube B (*OD* = 1.8 mm & *t* = 0.16 mm)	
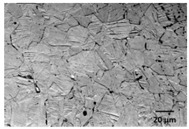		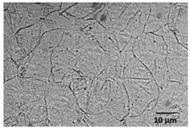	
%Al	2.819	%Al	2.608
%Zn	1.082	%Zn	1.023
%Si	0.052	%Si	0.036
%Ca	0.035	%Ca	0.039
%Cu	0.034	%Cu	0.0017
%Mn	0.016	%Mn	0.016
%Fe	0.012	%Fe	0.014
%Mg	95.80	%Mg	95.90

**Table 3:** Laser unit specifications IPG YLR-150/1500-QCW-AC.

Characteristic	Conditions	Unit
Operation mode	Continuous wave (CW)	-
Operation sub-mode	Modulated/rectangular waveform	-
Fiber core diameter	50	μm
Wavelength (λ)	1070	nm
Maximum peak power (CW with modulation)	250	W
Minimum pulse width (CW with modulation)	0.01	ms
Beam parameter product	1	mm × mrad
M^2^	2.82	-
Nozzle diameter	0.50	mm

**Table 4:** Process parameters for experimentation without gas blowing through the tube.

Tube	Process Parameter	Level Code				
		−1.414	−1	0	1	1.414
Tube A (*OD* = 3.0 mm & *t* = 0.22 mm)	Pulse Overlap, *O*_f_ (%)	19.65	30.00	55.00	80.00	90.36
	Pulse energy, *E*_p_ (mJ)	26.97	28.00	30.50	33.00	34.04
Tube B (*OD* = 1.8 mm & *t* = 0.16 mm)	Pulse Overlap, *O*_f_ (%)	7.99	20.00	49.00	78.00	90.01
	Pulse energy, *E*_p_ (mJ)	26.97	28.00	30.50	33.00	34.04

**Table 5:** Process parameters for experimentation with gas blowing through the tube.

Tube	Pulse Overlapping, *O*_f_ (%)	Pulse Energy, *E*_p_ (mJ)
Tube A (*OD* = 3.0 mm & *t* = 0.22 mm)	90.36	30.5
Tube B (*OD* = 1.8 mm & *t* = 0.16 mm)	78.0	33.0

**Table 6:** Applied process parameters on a laser cutting machine without gas blowing through the tube.

Tube	Parameter	Level Code				
		−1.414	−1	0	1	1.414
Tube A (*OD* = 3.0 mm & *t* = 0.22 mm)	Pulse frequency, *f* (Hz)	1000	1000	1100	1200	1200
	Cutting speed, *v*_f_ (mm/min)	1000	875	625	300	150
	Pulse Overlapping, *O*_f_ (%)	19.87	30.00	54.47	80.00	90.00
	Peak power, *P*_peak_ (W)	150	150	160	170	170
	Pulse width, *τ* (ms)	0.180	0.187	0.190	0.194	0.200
	Pulse energy, *E*_p_ (mJ)	27.00	28.05	30.40	32.98	34.00
Tube B (*OD* = 1.8 mm & *t* = 0.16 mm)	Pulse frequency, *f* (Hz)	600	600	700	800	800
	Cutting speed, *v*_f_ (mm/min)	690	600	450	220	100
	Pulse Overlapping, *O*_f_ (%)	7.85	20.00	48.49	78.00	90.00
	Peak power, *P*_peak_ (W)	150	150	160	170	170
	Pulse width, *τ* (ms)	0.180	0.187	0.190	0.194	0.200
	Pulse energy, *E*_p_ (mJ)	27.00	28.05	30.40	32.98	34.00

**Table 7:** Results with average *R_a_* and back-wall dross without gas blowing through the tube.

Trial	Tube							
	Tube A (*OD* = 3.0 mm & *t* = 0.22 mm)				Tube B (*OD* = 1.8 mm & *t* = 0.16 mm)			
	*O*_f_ (%)	*E*_p_ (mJ)	*R_a_* (μm)	*D*_bw_ (%)	*O*_f_ (%)	*E*_p_ (mJ)	*R_a_* (μm)	*D*_bw_ (%)
1	30.00	28.00	1.12	0.81	20.00	28.00	1.24	0.67
2	80.00	28.00	0.71	0.52	78.00	28.00	0.66	0.72
3	30.00	33.00	1.05	0.61	20.00	33.00	1.15	0.78
4	80.00	33.00	0.73	0.10	78.00	33.00	0.68	0.28
5	55.00	30.50	1.00	0.37	49.00	30.50	1.00	0.57
6	55.00	30.50	1.05	0.19	49.00	30.50	1.05	0.41
7	55.00	30.50	1.02	0.24	49.00	30.50	0.89	0.57
8	19.64	30.50	1.26	0.54	7.99	30.50	1.33	0.94
9	90.36	30.50	0.71	0.31	90.01	30.50	0.71	0.36
10	55.00	26.96	1.17	0.74	49.00	26.96	0.93	0.51
11	55.00	34.04	0.96	0.58	49.00	34.04	0.99	0.46
12	55.00	30.50	0.99	0.31	49.00	30.50	1.03	0.35
13	55.00	30.50	1.08	0.24	49.00	30.50	1.06	0.28
14	55.00	30.50	0.96	0.33	49.00	30.50	0.98	0.27

**Table 8:** ANOVA results for surface response methodology without gas blowing through the tube.

Tube	Tube A (*OD* = 3.0 mm & *t* = 0.22 mm)					Tube B (*OD* = 1.8 mm & *t* = 0.16 mm)			
Response	*R_a_*			*D*_bw_		*R_a_*		*D*_bw_	
R^2^	90.7			88.7		95.1		87.1	
Source	DF	SS	P	SS	P	SS	P	SS	P
Blocks	1	0.01	0.13	0.00	0.56	0.01	0.16	0.05	0.07
Regression	5	0.31	**0.00**	0.52	**0.00**	0.47	**0.00**	0.44	**0.01**
Linear	2	0.30	**0.00**	0.24	**0.01**	0.46	**0.00**	0.22	**0.01**
*O*_f_	1	0.28	**0.00**	0.16	**0.01**	0.46	**0.00**	0.20	**0.00**
*E*_p_	1	0.01	0.13	0.09	**0.02**	0.00	0.97	0.02	0.20
Quadratic	2	0.02	0.25	0.27	**0.00**	0.01	0.34	0.14	**0.02**
*O*_f_ ×*O*_f_	1	0.02	0.11	0.02	0.11	0.00	0.84	0.12	**0.01**
*E*_p_ × *E*_p_	1	0.00	0.80	0.25	**0.00**	0.01	0.15	0.02	0.23
Interaction	1	0.00	0.54	0.01	0.30	0.00	0.38	0.07	**0.03**
*O*_f_ × *E*_p_	1	0.00	0.54	0.01	0.30	0.00	0.38	0.07	**0.03**
Res. error	7	0.03	-	0.07	-	0.02	-	0.07	-
Lack of fit	3	0.02	0.14	0.05	0.18	0.01	0.62	0.05	0.15
Pure error	4	0.01	-	0.02	-	0.02	-	0.02	-
Total	13	0.36	-	0.59	-	0.51	-	0.56	-
